# Supplementary figures and images for: QuantTB – a method to classify mixed Mycobacterium tuberculosis infections within whole genome sequencing data
Source: BMC Genomics. 2020 Jan 28;21:80. doi: 10.1186/s12864-020-6486-3 (PMC6986090; doi:10.1186/s12864-020-6486-3)

Number of pairwise SNPs

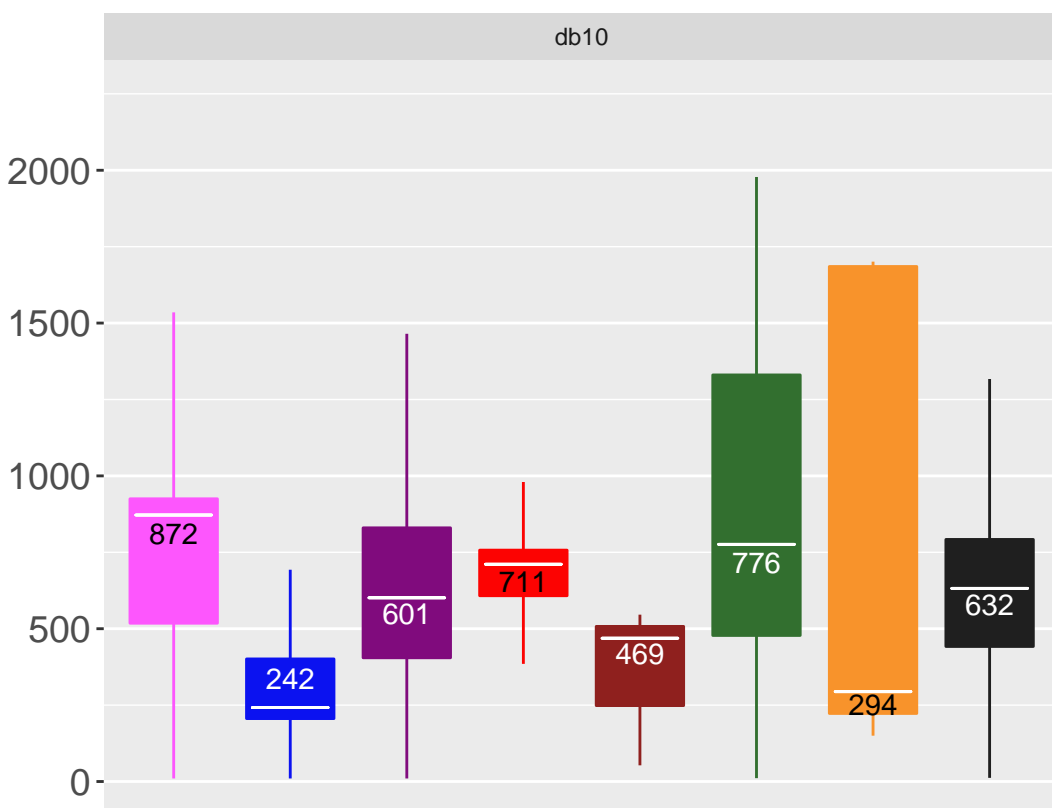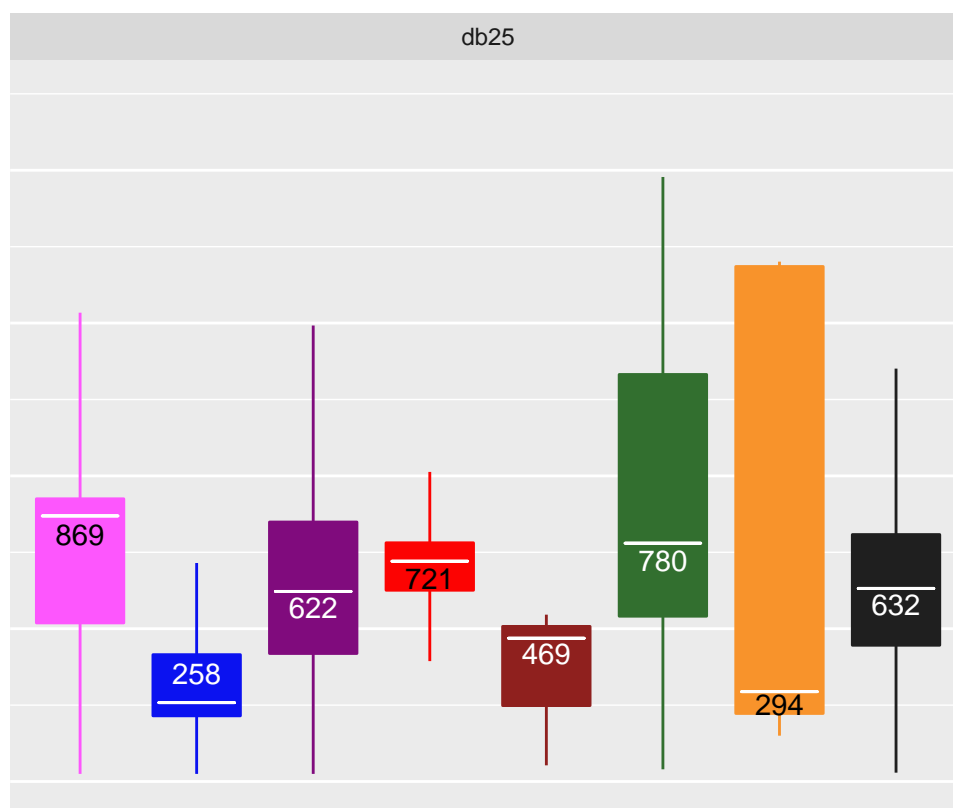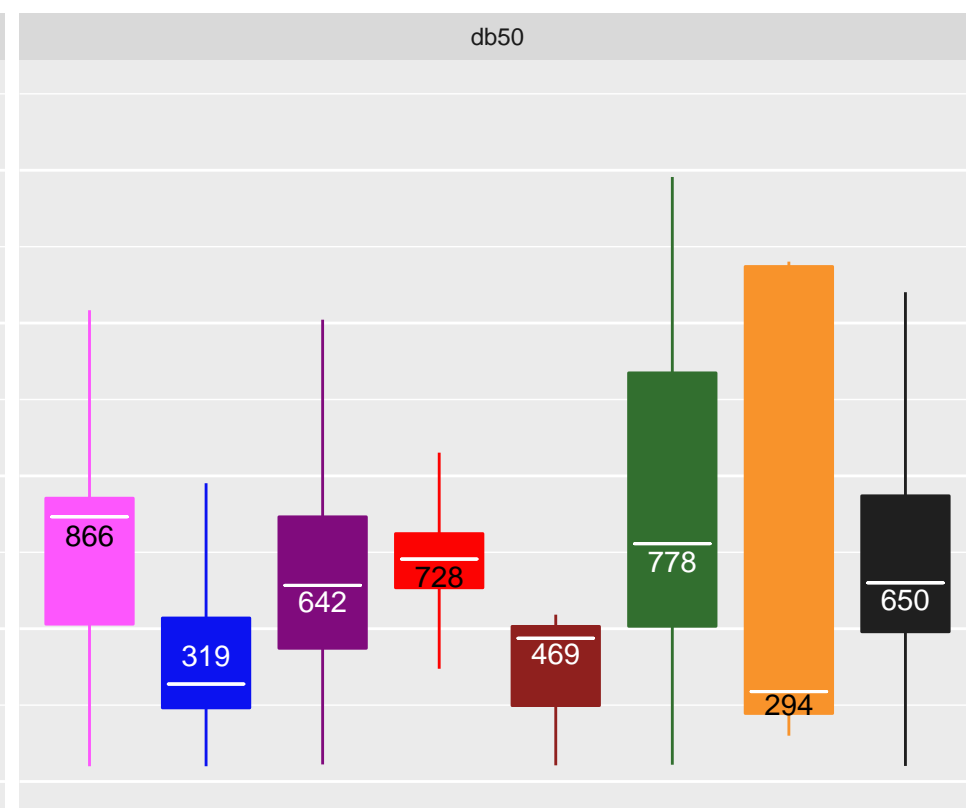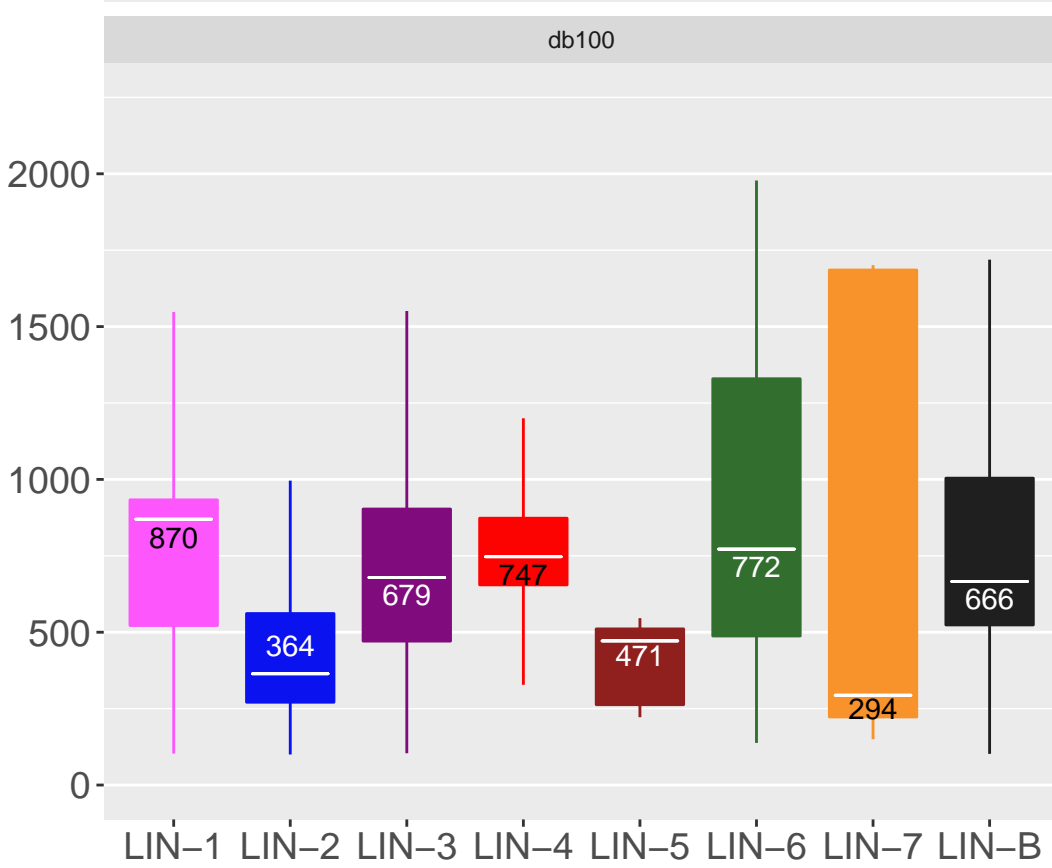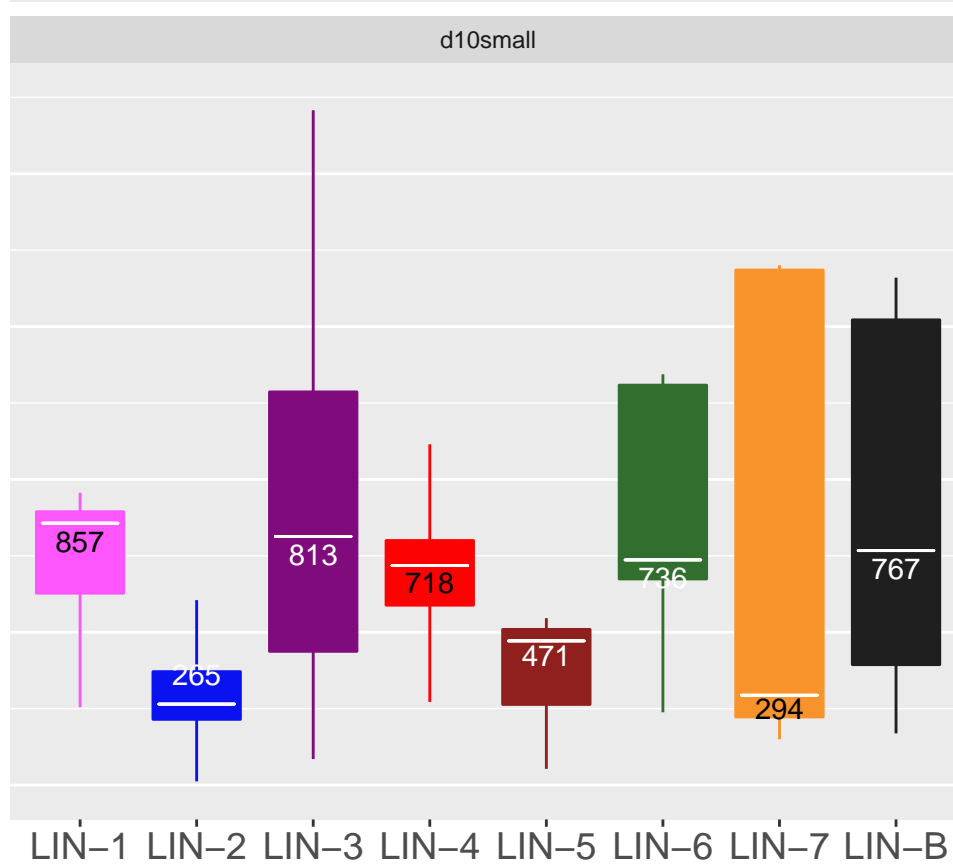

LIN-1 LIN-2 LIN-3 LIN-4 LIN-5 LIN-6 LIN-7 LIN-B

Supplement: Supplementary file 1 — Additional file 1: Figure S1. Intra-lineage pairwise distance for the different M. tuberculosis genomes in different databases. Pairwise distance was measured as the number of unique SNPs between a pair of genomes. The number in the box plot is the median distance of all pairs of samples from that lineage. The databases vary in the number of genomes and the minimum SNP distance between strains. [file 12864_2020_6486_MOESM1_ESM.pdf]

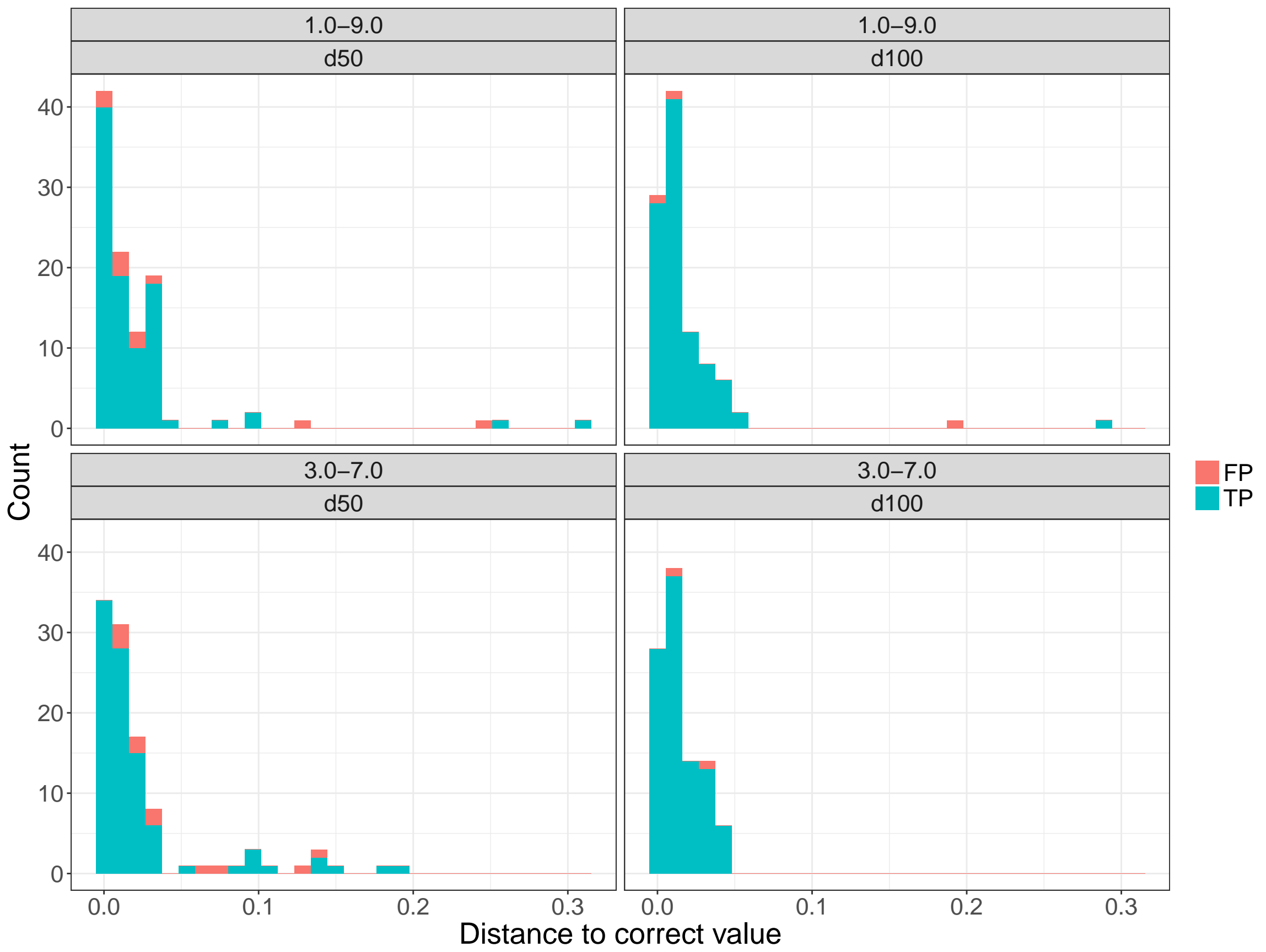

Supplement: Supplementary file 2 — Additional file 2: Figure S2. Histogram of residual error of QuantTB’s relative abundance prediction across different testing scenarios. The x axis is the difference between relative abundance prediction of QuantTB and the actual relative abundance value. The y axis is the total number of strains with that error. A distance of 0 means that QuantTB predicted the relative abundance perfectly for that strain. Higher distance/error indicates that QuantTB predicted the relative abundance further away from the actual value. Color indicates whether QuantTB predicted the correct strain (TP) or the wrong strain (FP). Tests were conducted using two different databases (d50 and d100) with mixed samples where the two strains are present at different coverages (1x-9x or 3x-7x). [file 12864_2020_6486_MOESM2_ESM.pdf]
